# Contextual loss based artifact removal method on CBCT image

**DOI:** 10.1002/acm2.13084

**Published:** 2020-11-02

**Authors:** Shipeng Xie, Yingjuan Liang, Tao Yang, Zhenrong Song

**Affiliations:** ^1^ College of Telecommunications and Information Engineering Nanjing University of Posts and Telecommunications Nanjing Jiangsu China

**Keywords:** CBCT, scatter correction, contextual loss

## Abstract

**Purpose:**

Cone beam computed tomography (CBCT) offers advantages such as high ray utilization rate, the same spatial resolution within and between slices, and high precision. It is one of the most actively studied topics in international computed tomography (CT) research. However, its application is hindered owing to scatter artifacts. This paper proposes a novel scatter artifact removal algorithm that is based on a convolutional neural network (CNN), where contextual loss is employed as the loss function.

**Methods:**

In the proposed method, contextual loss is added to a simple CNN network to correct the CBCT artifacts in the pelvic region. The algorithm aims to learn the mapping from CBCT images to planning CT images. The 627 CBCT‐CT pairs of 11 patients were used to train the network, and the proposed algorithm was evaluated in terms of the mean absolute error (MAE), average peak signal‐to‐noise ratio (PSNR) and so on. The proposed method was compared with other methods to illustrate its effectiveness.

**Results:**

The proposed method can remove artifacts (including streaking, shadowing, and cupping) in the CBCT image. Furthermore, key details such as the internal contours and texture information of the pelvic region are well preserved. Analysis of the average CT number, average MAE, and average PSNR indicated that the proposed method improved the image quality. The test results obtained with the chest data also indicated that the proposed method could be applied to other anatomies.

**Conclusions:**

Although the CBCT‐CT image pairs are not completely matched at the pixel level, the method proposed in this paper can effectively correct the artifacts in the CBCT slices and improve the image quality. The average CT number of the regions of interest (including bones, skin) also exhibited a significant improvement. Furthermore, the proposed method can be applied to enhance the performance on such applications as dose estimation and segmentation.

## 1 INTRODUCTION

Although cone beam CT has great potential in clinical applications, the challenge of scattered radiation decreases the image quality, leading to many artifacts in the images.^1^, ^2^, ^3^ Artifacts (including streaking, shadowing, ringing, and cupping artifacts, etc.) are generally defined as the difference between the reconstructed value of the CT image and the true distribution of attenuation coefficient of the object. In the published literature, the main correction methods can be divided into two types based on their different processing methods.^4^ One is a hardware processing method, which prevents the scattered rays generated during the attenuation process from reaching the detector to the greatest extent possible. Common methods include the air‐gap method, collimator method, filter method, antiscattering grating method, and the method employing a modulator.^5^, ^6^ However, the increase in hardware equipment introduces operational difficulties in the CBCT system and increases the cost of the entire process.^7^ The second type of correction method is digital image processing technology, which mainly estimates the scattering distribution via experiments, and then adopts postprocessing methods to suppress or eliminate scattering. Common methods include Monte Carlo simulation, scattering estimation‐based methods, and convolution model‐based methods.

The principle of the Monte Carlo simulation method is to find the scattering distribution by simulating the photon trajectory of the scattering event. For example, the method in 8 uses the precise physical model of PENELOPE to simulate photon transmission in a voxelized geometry. The method in 9 combines GPU‐based Monte Carlo (MC) simulation with patient CT images to present an ultrafast scattering correction framework, thereby achieving scattering correction and image reconstruction. The fast Monte Carlo simulation method proposed by Saucier et al^10^ and the optimized Monte Carlo simulation method proposed by Xun et al^11^ also achieved good artifact correction effects. However, Monte Carlo simulations caused huge time consumption and limited their clinical application,^12^, ^13^ so it needs to trade‐off between accuracy and simulation time. Information such as x‐ray spectral characteristics, object geometry and attenuation coefficient are critical for methods based on scattering estimation. Based on the above information, Yao et al. obtained an approximate estimate of the artifacts,^14^ Yang et al. could estimate the additional scattering from the shadow region,^1^ and Stankovic et al. used the hybrid scattering estimation model to generate the scattergram.^15^ Satisfactory results were also obtained using the level set^16^ and moving block^17^ methods. People have started paying attention to convolution‐based methods. For example, Zhao et al. introduced free parameters in the convolution kernel to identify the optimal parameters, so that the model of the scattering potential and the convolution kernel could best fit the approximate estimate of the scattering profile of the previously known image objects.^18^ Baer et al. incorporated physical scatter correction method in a convolution‐based scatter correction algorithm.^19^

Deep learning has become a popular method in the field of computer vision with the advantage of learning complex models end‐to‐end. Li et al^20^ proposed an encoder–decoder 2D U‐Net neural network for the CBCT correction. Its main idea is to use deep convolutional neural network (DCNN) to generate synthetic CT images. Xie et al. proposed the use of artifact‐free CNN (AFCNN) to correct scattering artifacts,^21^ where the mean squared error (MSE) was used as the loss function. This method combined a deep CNN and a residual learning framework (RLF) to train a mapping function from an uncorrected image to a corrected image. The CBCT image blocks were used as the input, whereas the CT image blocks were used as the label. The results showed that this method could effectively suppress artifacts in the CBCT images.

Generative adversarial networks (GANs) are widely used in image reconstruction. Kida et al^22^ developed a comprehensive method based on CycleGAN to generate synthetic CT images from CBCT images, which defined the content of bad mapping in a quantitative way in terms of a loss function, thereby finding an approximate map that minimizes the loss function. On the basis of CycleGAN model, Liang et al. integrated the adversarial loss, cycle consistency loss and identity mapping loss to convert CBCT into CT‐like images, and achieved a MAE of approximately 40 HU in the head and neck patient test cases.^23^ Kurz et al^24^ successfully trained a periodical generation adversarial network using unpaired training data to perform CBCT to CT image conversion, thereby correcting the CBCT intensity. Harms et al. introduced the concept of residual blocks into the cycle‐consistent adversarial network (CycleGAN) framework to understand the mapping between CBCT images and paired planned CT images.^25^

Inspired by the method proposed by Merchez et al,^26^ we added contextual loss to a simple five‐layer CNN network to correct the CBCT artifacts in the pelvic region. The loss function consists of two parts, $L_{\mathrm{CX}}^{t}$ and $L_{\mathrm{CX}}^{s}$. $L_{\mathrm{CX}}^{t}$ measures the loss of the generated image and label image, while $L_{\mathrm{CX}}^{s}$ measures the loss of the generated image and input image. Contextual loss plays a key role in the optimization of the CNN network performance. We chose to conduct this feasibility study in the context of pelvic CBCT images. We provide training data and ground truth data to the network for supervised machine learning.

The remainder of this paper is organized as follows. In Section II, we describe the method used. The experimental results are presented in Section III. Finally, the discussion and conclusions are reported in Section IV.

## 2 materials and methods

**Fig. 1:**
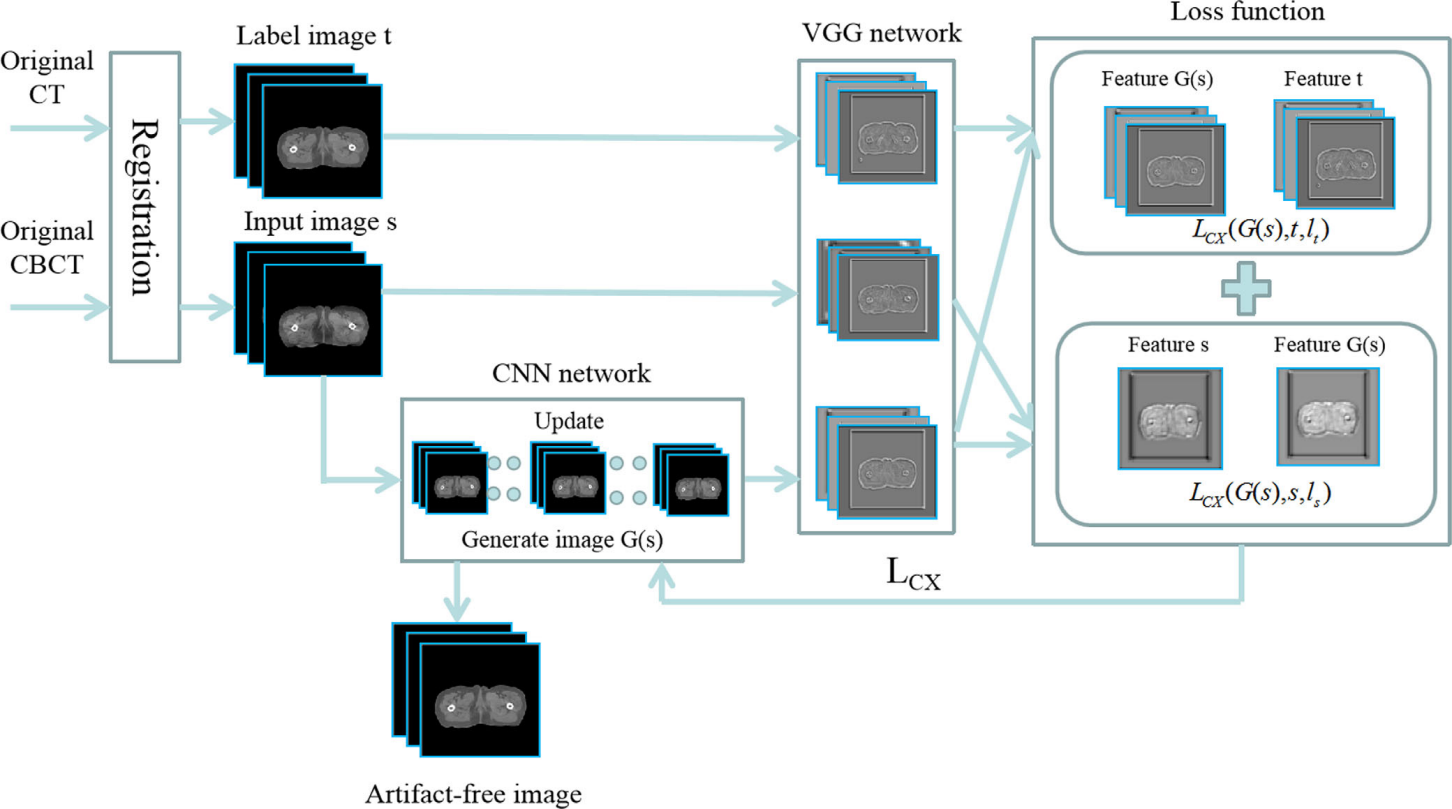
Experimental process.

The experimental method in this paper can be briefly summarized as shown in Fig. 1. Next, we will introduce each part of Fig. 1 in detail.

### 2.1 Registration

According to Fig. 1, the registration is first performed after obtaining the original data. Image registration involves aligning an analysis image with a reference image using a geometric transformation that correlates these two images. Medical image registration methods can be broadly categorized into rigid and nonrigid registrations. Nonrigid registration is widely applied to deal with large motion and interfraction variability in chest and abdomen. In this study, we used the nonrigid grid registration method.

Organ movements might be obvious in IGRT. For example, pelvic anatomy, which includes the prostate and rectum, could change during IGRT.^27^, ^28^ To prevent the significant difference between the CBCT‐CT training pairs from affecting the experimental results, we referred to the method detailed in 29 to perform deformable image registration (DIR) on the pelvic region and subsequently generated the CBCT‐CT training pairs required for the experiment. The CBCT image is static and the CT image is moving during DIR. The structural differences between the CBCT‐CT training pairs can be reduced via deformable registration.

It should be noted that the number of CBCT slices and CT slices and slice thickness in the original data are different. Although the algorithm proposed in this paper can be applied to misaligned data, it is also crucial for the medical images to retain quantitative image values. Therefore, a certain registration is necessary to match the patient's data before the data are input to the network. In this step, we mainly introduced DIR technology to correspond to the slices and adjusted the parameters, and there was no large deformation. However, mismatches still exist between the CBCT‐CT training pairs following registration.

### 2.2 Data set

As the training of the convolutional networks is inseparable from data set, the generation of datasets is related to whether the trained model can sufficiently represent all the sample spaces. We used the patient pelvis data for training in the proposed clinical application method. Specifically, patients were required to undergo a CT scan of the pelvic region before the start of IGRT. In the subsequent radiotherapy, the patients underwent a CBCT scan so that the pelvic area could be monitored in real time. Therefore, our data were CBCT images and CT images that were obtained from an IGRT system.

The original data came from 11 patients. The size of the CT images is 512 × 512, while CBCT data are composed of six groups of 384 × 384 and five groups of 512 × 512. Considering the small sample size, we used data expansion techniques in the experiment, such as image rotation, and obtained 2179 CBCT slices and 2036 CT slices. For CBCT, the slice thickness is usually 3.0 mm, and the pixel size is $0.8789\times 0.8789\;{\text{mm}}^{2}$. However, for CT, the slice thickness is displayed as 2.5mm or 3.0mm, and the pixel size is $0.9766\times 0.9766\;{\text{mm}}^{2}$.

The above original images were preprocessed by DIR to generate 627 CBCT‐CT pairs, which was the data set used in our experiment. It should be pointed out that the data set is a 2D data set. During the training process, we randomly selected 499 pairs of CBCT and CT images as the training set, 64 pairs as the validation set and 64 pairs as the test set. The size of each image was 512 × 512. Among them, the CBCT images were used as the input images, whereas the CT images were used as the label images. However, a complete correspondence between the registered CBCT‐CT pairs was still not achieved, and the slightly misplaced input‐tag image pairs rendered the pixel wise loss function unsuitable for training.

### 2.3 Contextual loss

The contextual loss function has excellent application prospects with respect to the slight misalignment of data. The main idea behind this function is that it assumes the image as a collection of features, and then determines the similarity between the images by measuring the similarity between the features. This loss function allows the local deformation of the image to a certain extent, therefore the requirement for the data to be aligned at the pixel level is moderate. In addition, the loss function used in this study constrains the local features, which enables it to operate on the region with similar semantics. Specifically, it first finds similar features in these regions with similar semantic meanings and forms a match between these features. The context of the entire image is then integrated, and the similarity between the images is represented by the similarities between the matching features. Therefore, we can categorize this process into the following:

#### 2.3.1 Feature extraction

As shown in Fig. 1, the input image (CBCT image) was sent to the CNN network to obtain a preliminary generated image. Next, the input image, generated image, and label image (three‐dimensional) were sent to the VGG19 network (proposed by Oxford's Visual Geometry Group) for feature extraction.

**Fig. 2:**
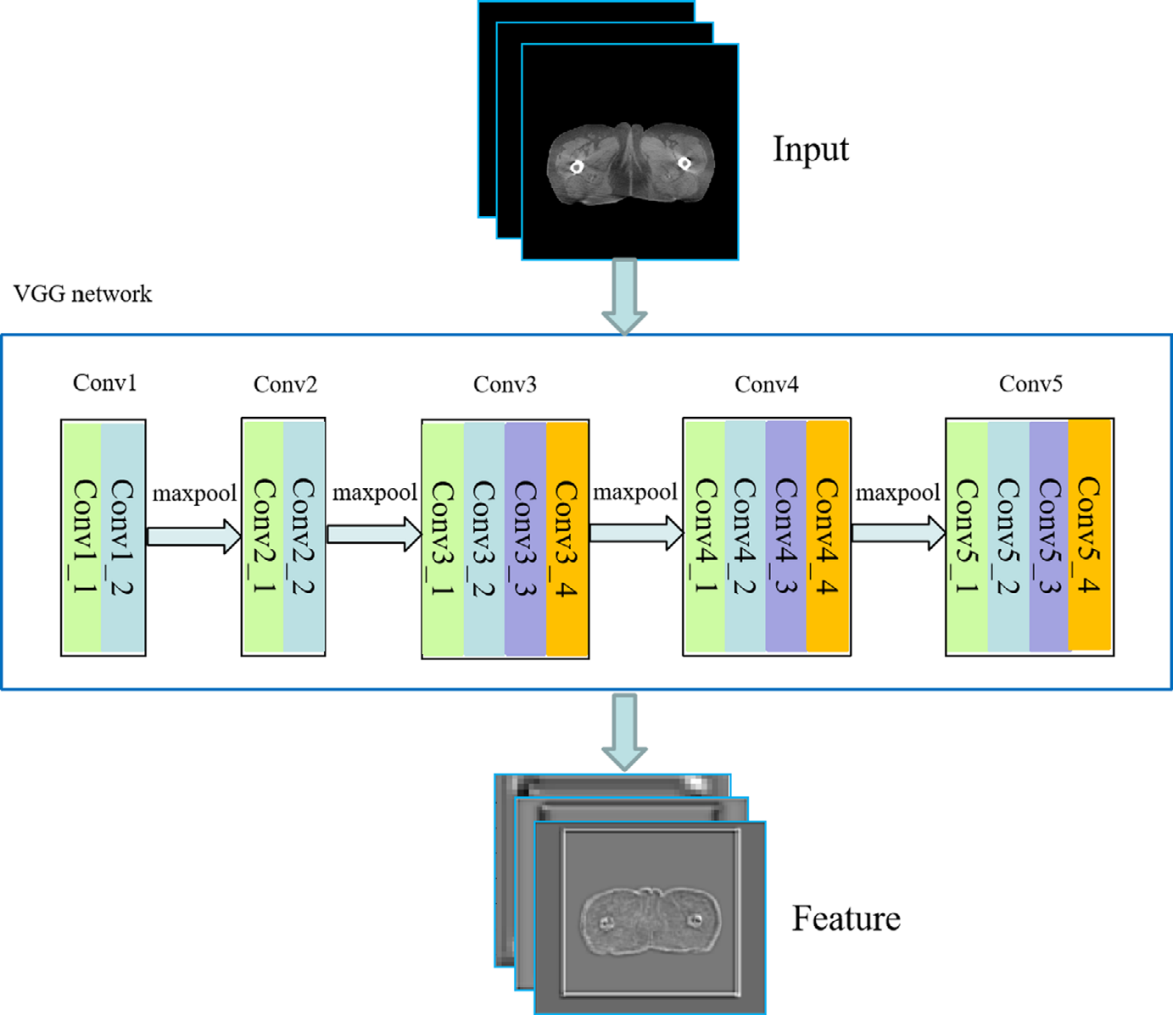
Network structure of VGG19.

In this study, we used the VGG19 network that was pretrained on ImageNet^30^ as the extractor. The pretrained VGG network takes three channels images as the input, while the CT images are grayscale images. Therefore, we duplicated the CT images into three channels before feed them into the VGG network. The VGG‐19 network contains 16 convolutional layers, followed by 3 fully connected layers. The features of the corresponding convolutional layers that were used to calculate the loss function will be described later. The VGG19 network structure used in this study is shown in Fig. 2.

Let the source image *s* and target image *t* be the two images to be compared, and *s_i_* and *t_j_* are the features obtained after the source image *s* and target image *t* are passed through VGG19, respectively. Then, we can represent each image as a set of features, namely S ={*s_i_*} and T = {*t_j_*}. Furthermore, we assume |S| = |T| = N, and when |S| ≠ |T|, N‐sampling is performed from a larger set. N represents the number of high‐dimensional points (features).

#### 2.3.2 Similarity between features

Next, we present a detailed introduction from a mathematical perspective to define the similarities between the features. Contextual loss is a loss function related to the cosine distance. Let $d_{\mathrm{ij}}$ denote the cosine distance between features, expressed as follows:

(1) $$d_{\mathrm{ij}}=\left(1‐\frac{\left(s_{i}‐\mu_{t}\right)\cdot \left(t_{j}‐\mu_{t}\right)}{{∥s_{i}‐\mu_{t}∥}_{2}{∥t_{j}‐\mu_{t}∥}_{2}}\right)\;\text{where,}\;\mu_{t}=\frac{1}{N}\sum_{j}t_{j}$$

when $d_{\mathrm{ij}}<<d_{\mathrm{ik}},\;\forall k\neq j$, we assume that features *s_i_* and features *t_j_* have similar contexts. To simplify the calculation, the cosine distance is normalized as follows:

(2) $$\overset{∼}{\underset{\mathrm{ij}}{d}}=\frac{d_{\mathrm{ij}}}{\min_{k}d_{\mathrm{ik}}+\varepsilon }$$

Here, we fixed ε=1e‐5. Using an exponential operation, we transformed the distance into similarity. The definition can be expressed as follows:

(3) $$w_{\mathrm{ij}}=\exp\left(\frac{1‐\tilde{d_{\mathrm{ij}}}}{h}\right)$$

where, h>0 is a bandwidth parameter. Here, we fixed h=0.5. Finally, we used a scale‐invariant version of the normalized similarity to define the contextual similarity between the features:

(4) $$CX_{\mathrm{ij}}=\frac{w_{\mathrm{ij}}}{\sum_{k}w_{\mathrm{ik}}}$$

#### 2.3.3 Similarity between images

**Fig. 3:**
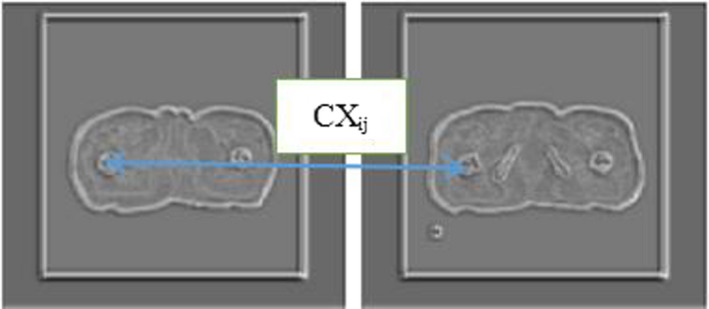
Feature matching.

We find the features *s_i_* that is most similar to features *t_j_* to form a match between the features, as shown by the arrows in Fig. 3, and the contextual loss can be regarded as the weighted sum of the arrows. The ratio of the above‐defined methods to the distance is robust. If *s_i_* is not similar to *t_j_*, *CX_ij_* will be low regardless of the distance between *s_i_* and *t_j_*. However, if the features *s_i_* and *t_j_* are similar, *CX_ij_* will be high even if they are not in the corresponding positions. We consider a pair of images to be similar when most features of one image can find similar features in another image.

We can mathematically define the contextual similarity between the images as follows:

(5) $$CX\left(s,t\right)=CX\left(S,T\right)=\frac{1}{N}\sum_{j}\max_{i}CX_{\mathrm{ij}}$$

where, $CX_{\mathrm{ij}}$ represents the similarity of the features $s_{i}$ and $t_{j}$. When an image is compared with itself, the feature similarity value is $CX_{\mathrm{ii}}=1$, which indicates that CX(S,S)=1. In contrast, when the feature set in one image differs completely from that in the other image, the feature similarity value is $CX_{\mathrm{ij}}=\frac{1}{N}\;\forall i,j$, indicating that $CX\left(S,T\right)\approx \frac{1}{N}\to 0$.

#### 2.3.4 CX loss function

In summary, the loss function can be expressed as follows:

(6) $$L_{\mathrm{CX}}\left(s,t,l\right)=‐\log\left(CX\left(\varphi^{l}\left(s\right),\varphi^{l}\left(t\right)\right)\right)$$

where φ represents the VGG19 network, and $\varphi^{l}\left(s\right),\varphi^{l}\left(t\right)$ represent the feature maps of the images s and t extracted from the layer l of network φ, respectively.

### 2.4 The proposed loss function

We trained a network G to map the given source image s to the output image G(s). Here, for network G, we used a five‐layer CNN network with adaptive dimensions. When the input image width≥128, the dimension was set to dim=512, else the dimension was dim=1024. In this experiment, the input image size was 512 × 512, so the initial width==512. Then the width was down‐sampled by width//2 until width==4, and the input image is loaded. The loss $L_{\mathrm{CX}}\left(G\left(s\right),t,l\right)$ represented the degree of similarity between the generated and target images, whereas the loss $L_{\mathrm{CX}}\left(G\left(s\right),s,l\right)$ was used to measure the similarity between the generated and source images. The loss function used in the experiment can be defined as follows:

(7) $$L\left(G\right)=L_{\mathrm{CX}}\left(G\left(s\right),s,l_{s}\right)+\lambda \cdot L_{\mathrm{CX}}\left(G\left(s\right),t,l_{t}\right)$$

where, $l_{s}=conv4\_2$ yields the content feature, and $l_{t}={\left(\left\{convk\_2\right)\right\}}_{k=2}^{4}$ yields the style feature. In the experiment, we randomly sampled the layer conv2_2 into 80 × 80 features to obtain better results, while reducing the required computational memory. We discovered that the difference in the number of randomly sampled features may be critical to the experiment. The specific analysis will be provided later. Here, λ is a constant that controls the ratio of the two loss functions. We set λ=5 in the experiments. It is noteworthy that the parameters above were obtained through multiple experiments and were found suitable for the experiments discussed in this study.

### 2.5 Network training

**Fig. 4:**
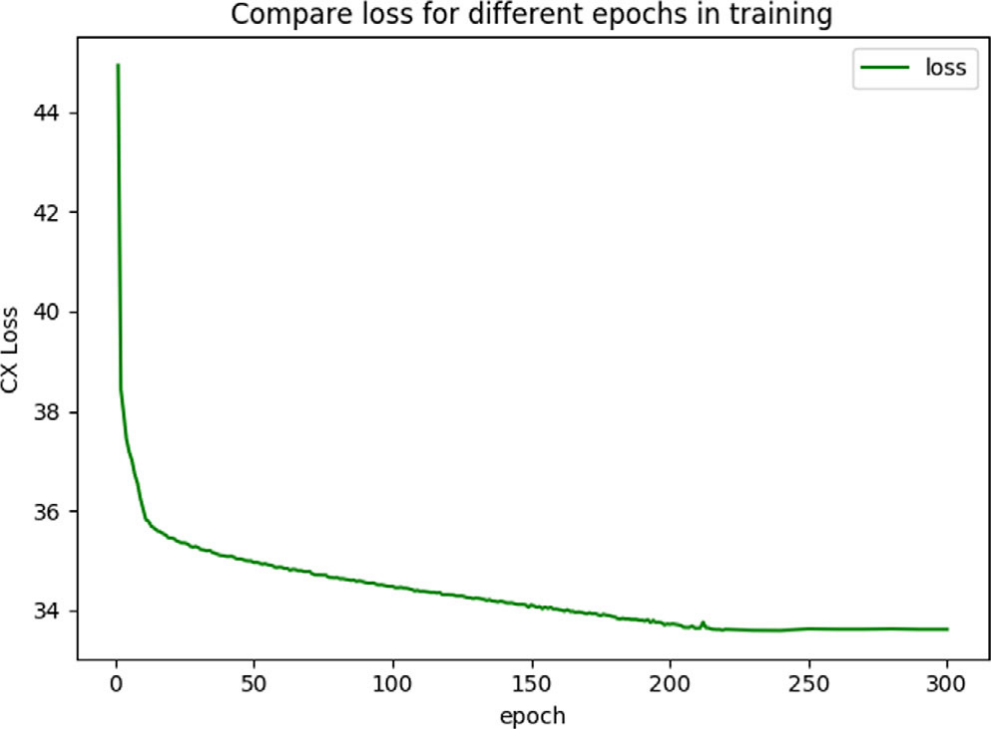
Change in training loss for different epochs.

The purpose of the training network was to obtain a mapping from the CBCT images to the planning CT images, which can improve the quality of the input CBCT images. First, a five‐layer CNN network was used to obtain the generated image G(s). When training the network, the loss was calculated according to the characteristics of the corresponding convolutional layer, and the image value of the reconstructed image G(s) was updated according to the change of the loss function. During the experimental process, image reconstruction was performed by iterative optimization. Changes in the loss function value and convergence during model training can objectively reflect the overall training effect of the model. The relationship between training loss and epoch is shown in Fig. 4. Based on the situations, the network parameters, feature sample size, and ratios of the two loss functions $L_{\mathrm{CX}}^{t}$ and $L_{\mathrm{CX}}^{s}$ were adjusted accordingly, and the training was repeated until the artifacts were effectively corrected.

We used the TensorFlow library in the Python environment on a GeForce GTX 1080 Ti processor. Adam optimizers and the nonlinear activation function ReLU were used in the experiment. Following the normal practice adopted in the deep learning community,^31^ each convolutional layer employed a small 3 × 3 kernel. We set the learning rate to 1e‐4 during the experiment. The number of epochs was set to 300, and the input–output image sizes were set to 512 × 512. The step size was set to 2 to achieve an accurate convergence.

### 2.6 Evaluation

The main difference between the method proposed in this paper and the method in 21 is that we introduced contextual loss, but method in 21 chose MSE loss. Perceptual loss can be applied to networks with mismatched data, Kupyn et al^32^ presented DeblurGAN network to reconstruct the image, which is based on conditional GAN and perceptual loss. We compared the proposed method with the above two methods. In the results section, we use pelvic data for statistical and visual analysis.

We also used other loss functions for comparison, such as L2 loss and perceptual loss. The formula is expressed as follows.

(8) $$L_{2}\left(x,y\right)={∥x‐y∥}_{2}$$

(9) $$L_{p}\left(x,y,l_{p}\right)={∥\varphi^{l_{p}}\left(x\right)‐\varphi^{l_{p}}\left(y\right)∥}_{1}$$

where φ represents the VGG19 network, and $\varphi^{l}\left(x\right),\varphi^{l}\left(y\right)$ represent the feature maps of the images x and y extracted from the layer l of network φ, respectively.

In this study, we calculated the mean absolute error (MAE), peak signal‐to‐noise ratio (PSNR), structural similarity (SSIM), and average CT number to quantify the results.

MAE is defined as the difference between the evaluation image and the CT image. The formula is expressed as follows:

(10) $$MAE=\frac{1}{m\times n}\sum_{i,j}^{m\times n}\left|y\left(i,j\right)‐\overset{\Lambda }{y}\left(i,j\right)\right|$$

where, *m* × *n* is the total number of pixels. *y*(*i*, *j*) is the value of the CT image with pixels (i, j), and $\overset{\Lambda }{y}\left(i,j\right)$ is the value of the evaluation image with pixels (i, j).

The input of PSNR was $\left(t,G(s)\right)$, where t and G(s) are the target and predicted images, respectively. The PSNR formula can be expressed as follows:

(11) $$PSNR=10\cdot \log_{10}\left(\frac{{\left(2^{n}‐1\right)}^{2}}{\mathrm{MSE}}\right)$$

where n is the number of sampling points. The number of sampling points in the natural image is 8, and the maximum pixel value is 255. The pixel range of the medical image is larger, and the corresponding n value needs to be adjusted for calculation.

Structural similarity (SSIM) is an index to measure the similarity of two images. The formula is expressed as follows.

(12) $$SSIM\left(x,y\right)=\frac{\left(2\mu_{x}\mu_{y}+c1\right)\left(2\sigma_{\mathrm{xy}}+c2\right)}{\left(\mu_{x}^{2}+\mu_{y}^{2}+c1\right)\left(\sigma_{x}^{2}+\sigma_{y}^{2}+c2\right)}$$

where *µ_x_* is the average of *x* and *µ_y_* is the average of y. $\sigma_{x}^{2}$ is the variance of *x*, $\sigma_{y}^{2}$ is the variance of *y*, and *σ_xy_* is the covariance of *x* and *y*. $c1={\left(k_{1}L\right)}^{2}$, $c2={\left(k_{2}L\right)}^{2}$ are constants used to maintain stability. *L* is the dynamic range of pixel values. $k_{1}=0.01$ and $k_{2}=0.03$.

The standard deviation represents the dispersion degree of pixel gray values relative to the mean. The larger the standard deviation, the more scattered the gray level distribution and the better the image quality.

(13) $$std=\frac{1}{m\times n}\sum_{i=1}^{m}{\sum_{j=1}^{n}\left(y(i,j)‐u\right)}^{2}$$

where, m×n is the total number of pixels. *y*(*i*, *j*) is the value of the evaluation image with pixels (*i*, *j*) and *u* stands for mean.

The average CT number can be obtained using the analysis measurement function of ImageJ software. Using the cursor to accurately select the area of interest, the system will give the CT number corresponding to that area.

## 3 results

In this study, we not only compared the artifact removal performance with other methods but also showed the process of finding the best performing network and parameters.

### 3.1 Experimental results

**Fig. 5:**
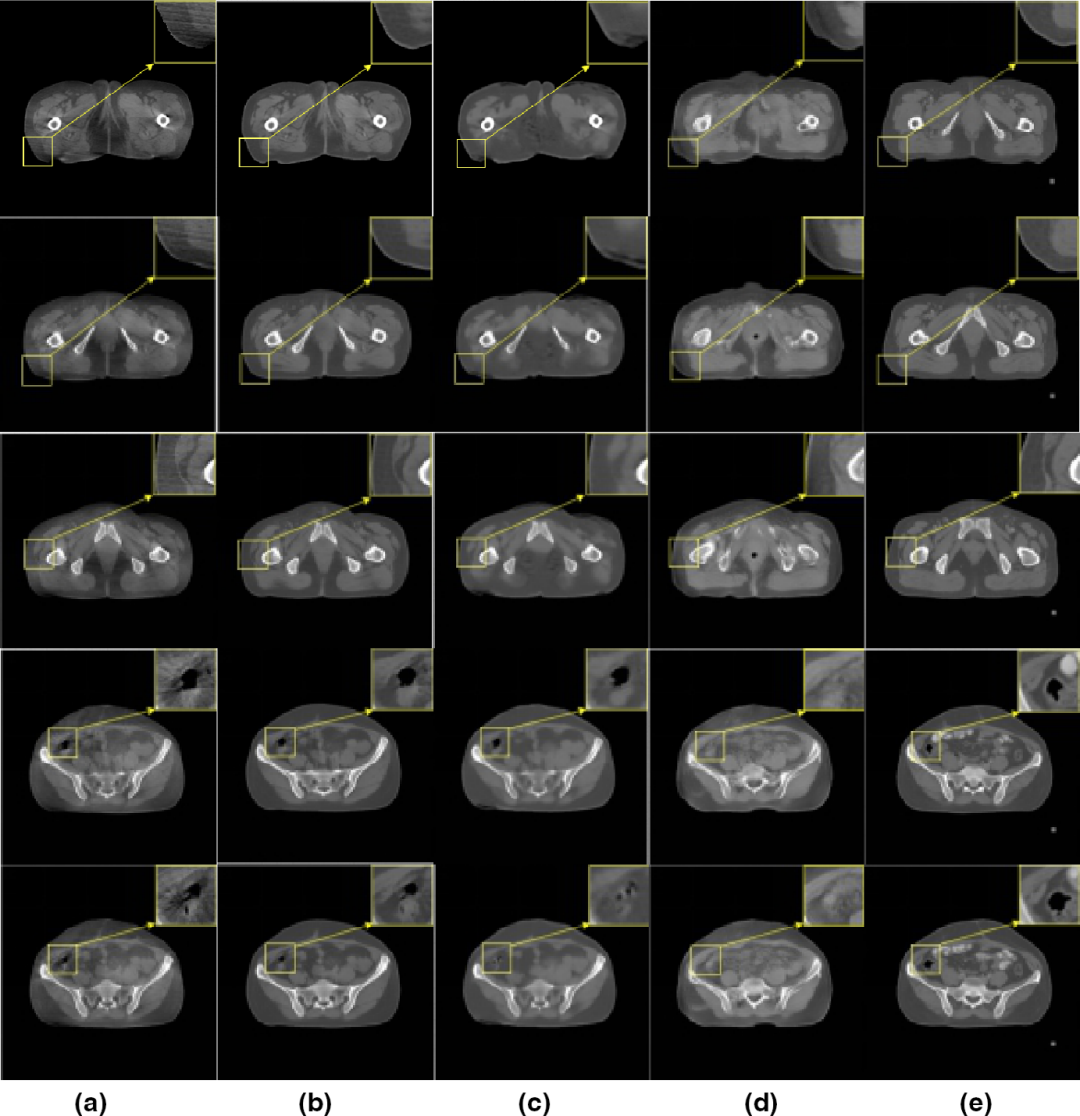
Artifact removal results obtained with the pelvis data. (a) CBCT, (b) proposed correction, (c) method in 21, (d) method in 32, and (e) RCT. Display window [−360, 628].

CBCT slices may be heavily contaminated with streak artifacts during the scanning process, which means that some detailed information may be destroyed. The proposed method effectively suppressed scattering artifacts in the CBCT slices, as indicated by the results shown in Fig. 5. For a clear comparison, the last column contains the corresponding CT images with few artifacts (RCT). Comparing Fig. 5a and Fig. 5b, it can be seen that the proposed method can correct artifacts in CBCT slices (including streaking, shadowing, ringing, cupping artifacts, etc.), which significantly improves the image quality. Observe the last line of Fig. 5b and Fig. 5c, although the CBCT slices processed by the method detailed in 21 also improved the quality of the slices, it introduced blurring during the smoothing correction process, making some details in the image lost. For example, it can be clearly seen that the reconstruction effect of the air cavity part was unsatisfactory. As shown in Fig. 5d, the method in 32 corrected the CBCT slice with poor performance. For example, the pubic bone on third row of d almost disappeared. In contrast, the proposed method preserved key details such as the internal contour and texture information of the pelvic region. Moreover, it is evident from Fig. 5b that the body contour of the CBCT image corrected using the method proposed herein was similar to that in the original CBCT image rather than that in the planning CT. This is a critical aspect in the IGRT workflow, as the body outline shows the patient's true position on the treatment table.

To objectively illustrate the effectiveness of our method in removing artifacts, a quantitative analysis (including CT number, MAE, PSNR, SSIM, std) of the pelvic region is presented in Table 1. Each of these analyses was derived from the mean values calculated over the test dataset. We calculated the CT numbers (in HU) of areas such as the bone marrow and skin; subsequently, we compared these values with the CT numbers in the original CT slices. It can be seen from Table 1 that compared with the methods in 21 and 32, the CT numbers of the slices corrected with the proposed method in the regions of interest above were closer to those of the original CT images. MAE dropped from 51.0124 to 46.0143 and standard deviation rose from 468.6564 to 483.3145 over the entire image. From Table 1, the standard deviation of skin is 346.1466 and the standard deviation of bone marrow is 182.0563. The PSNR values of the CBCT images may exceed 23.0696 dB and the SSIM values reached 0.8873. These indicate that our method improved the image quality and the artifacts were effectively suppressed by the well‐trained CNN.

**Table 1:** Quantitative analysis of the pelvis

Measurement	CBCT	CT	Proposed method	Method in 21	Method in 32
Mean CT numbers (HU)					
Bone marrow	220.2080	232.3675	226.8863	222.4910	85.4
Skin	−186.091	−140.134	−148.373	−185.189	−160.9
MAE (HU)	51.0124	/	46.0143	48.3359	75.8857
Standard Deviation of MAE (HU)	5.3769	/	5.2783	5.3164	5.4567
Average Standard deviation of images (HU)					
Whole image	468.6564	485.0308	483.3145	470.2253	395.7188
Bone marrow	170.7348	189.6158	182.0563	173.0177	117.5446
Skin	324.6481	382.8895	346.1466	310.6993	331.8619
Average PSNR	22.6595	/	23.0696	19.5833	11.4084
Average SSIM	0.8749	/	0.8873	0.8667	0.7493

**Fig. 6:**
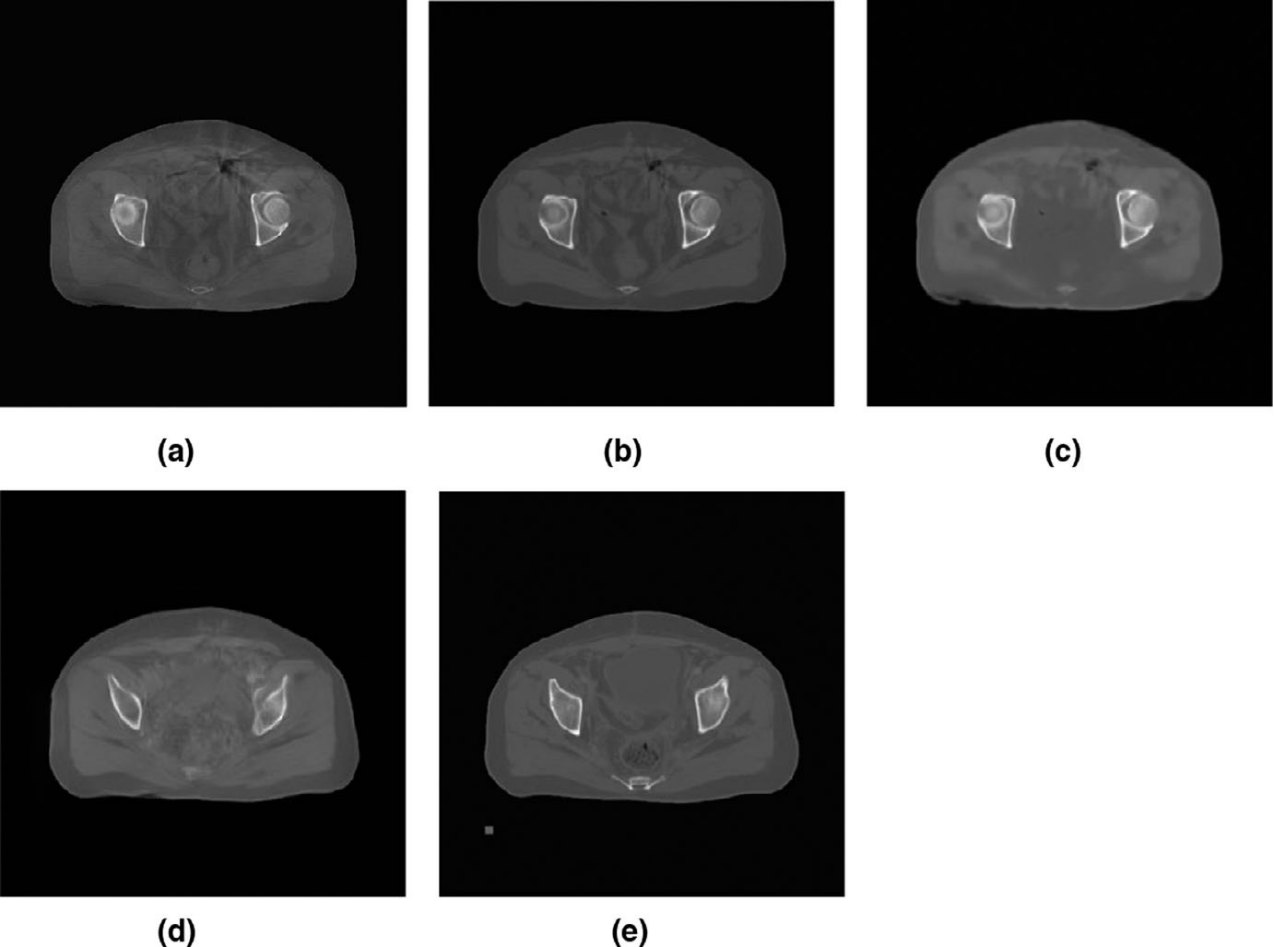
Artifact removal results with different loss function. (a) CBCT, (b) Contextual loss, (c) L2 loss, (d)Perceptual loss, and (e) RCT. Display window [−502, 528].

Fig. 6 shows the experimental results of using different loss functions to remove artifacts. Table 2 and Table 3, respectively, show the MAE and SSIM values of several different slices. The results show that the proposed loss function improves the network performance.

**Table 2:** MAE (HU) values of different slices and loss functions

	L2 loss	Perceptual loss	Contextual loss
Case 1	45.2318	68.5743	41.5959
Case 2	40.8850	63.3398	37.4898
Case 3	34.4598	60.6695	32.1762

**Table 3:** SSIM values of different slices and loss functions

	L2 loss	Perceptual loss	Contextual loss
Case 1	0.9030	0.6820	0.9048
Case 2	0.9046	0.7367	0.9083
Case 3	0.9048	0.7667	0.9073

**Fig. 7:**
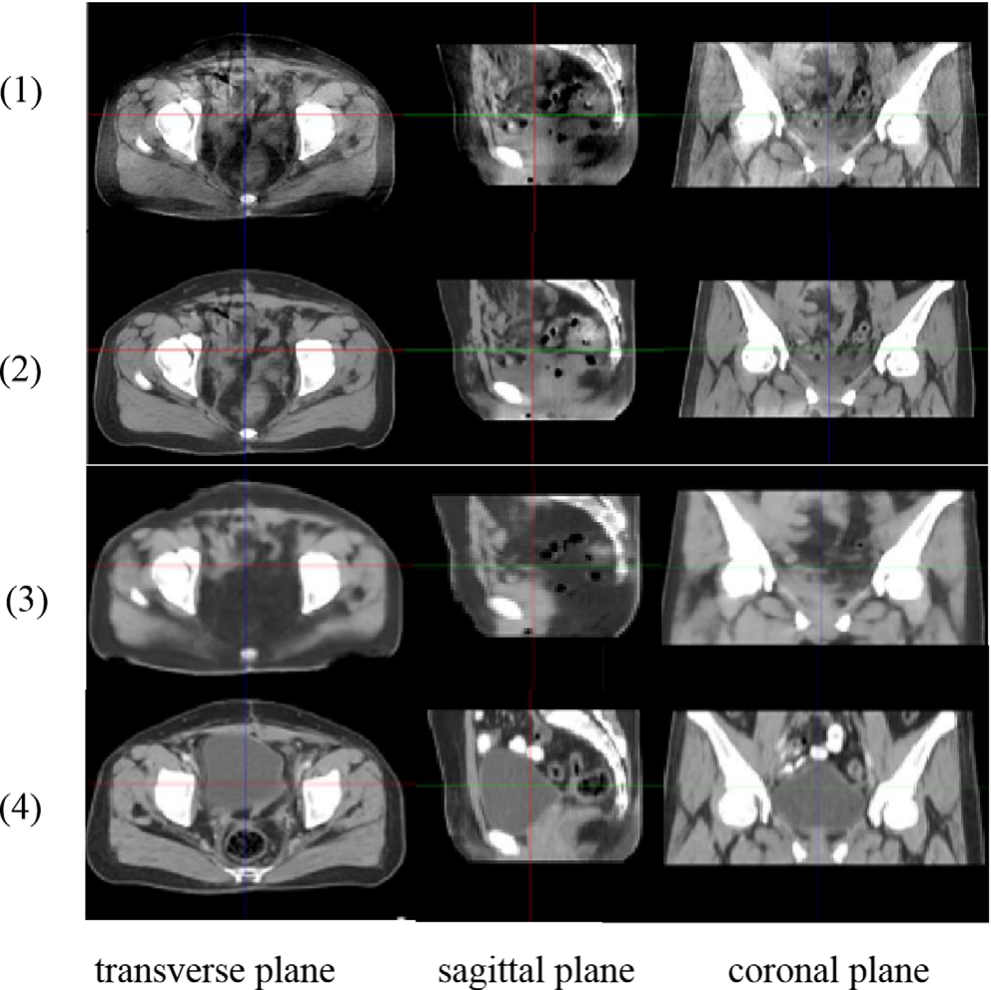
(1) CBCT image with scatter artifact, (2) Artifacts removal image obtained by the proposed method, (3)Artifacts removal image obtained by the method in 21, (4) RCT. Display window is [−160, 240].

Images of the transverse, coronal, and sagittal planes are shown in Fig. 7. As can be seen from Fig. 7, the three cut planes of the input image exhibited clear streak and cupping artifacts, which significantly reduced the image quality. Our method effectively retained the edge information of the image, while removing numerous artifacts in the image. According to Fig. 7, the quality of the resulting image obtained by our method was similar to that of a planning CT image.

**Fig. 8:**
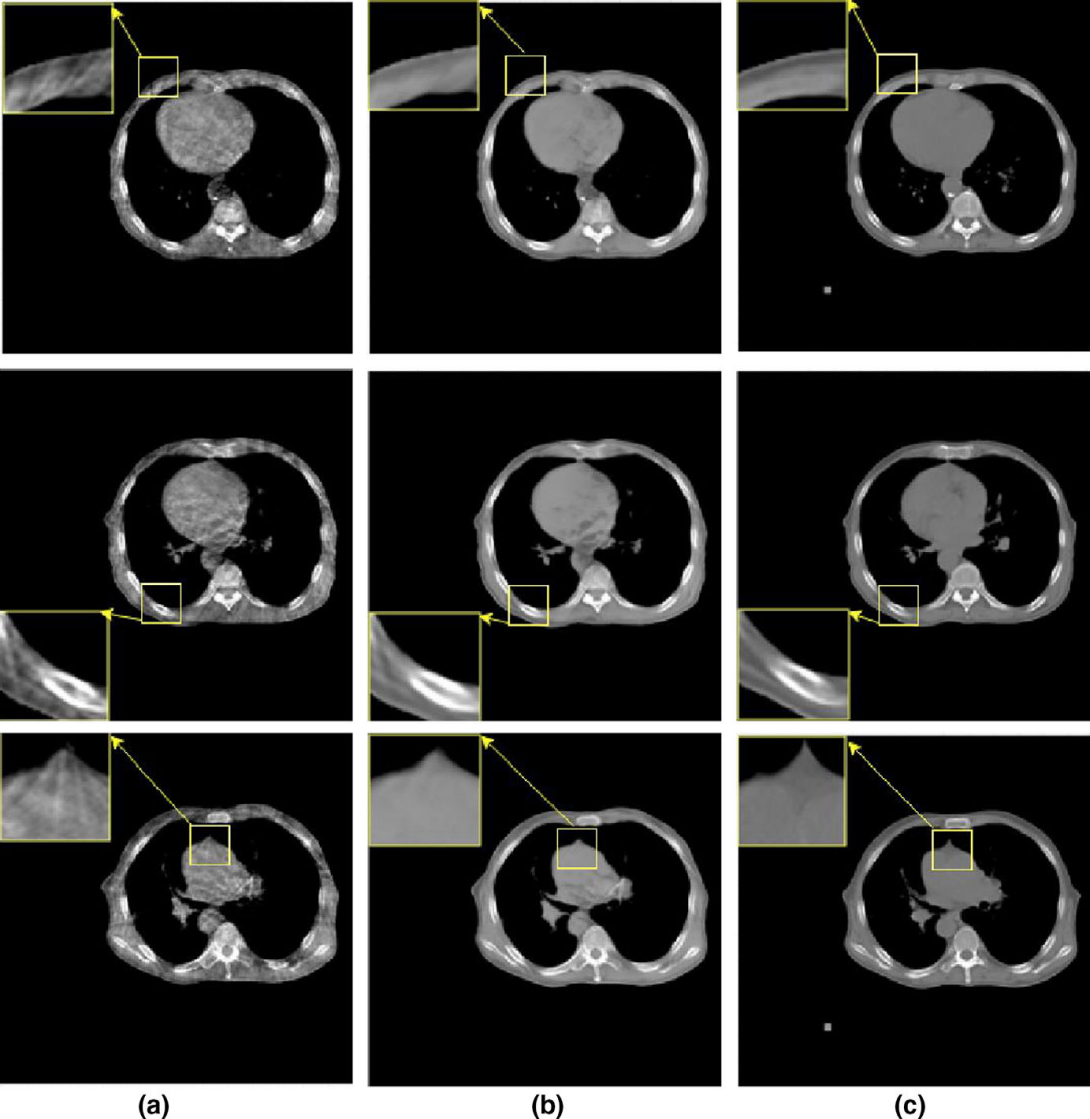
Artifact removal results obtained with the chest data. (a) CBCT, (b) proposed correction, and (c) RCT. Display window [−401, 526].

Breathing and other movements were more significant in the chest region than they were in the pelvic region. To verify that our method is applicable to other anatomies, thoracic data were input to the network for training. Specifically, we obtained 1,225 CT images and 1,093 CBCT images from the hospital. In these original data, CT images are all 512 × 512, while CBCT data are composed of 12 groups of 384 × 384 and 8 groups of 512 × 512. After these original images are preprocessed by the 3D registration system, 1225 CT‐CBCT pairs are generated. Randomly select 64 pairs as the verification set and 64 pairs as the test set for the experiment. Fig. 8 shows that good results were achieved even with the chest data. Here, we only selected three slices for display.

### 3.2 Optimization

**Fig. 9:**
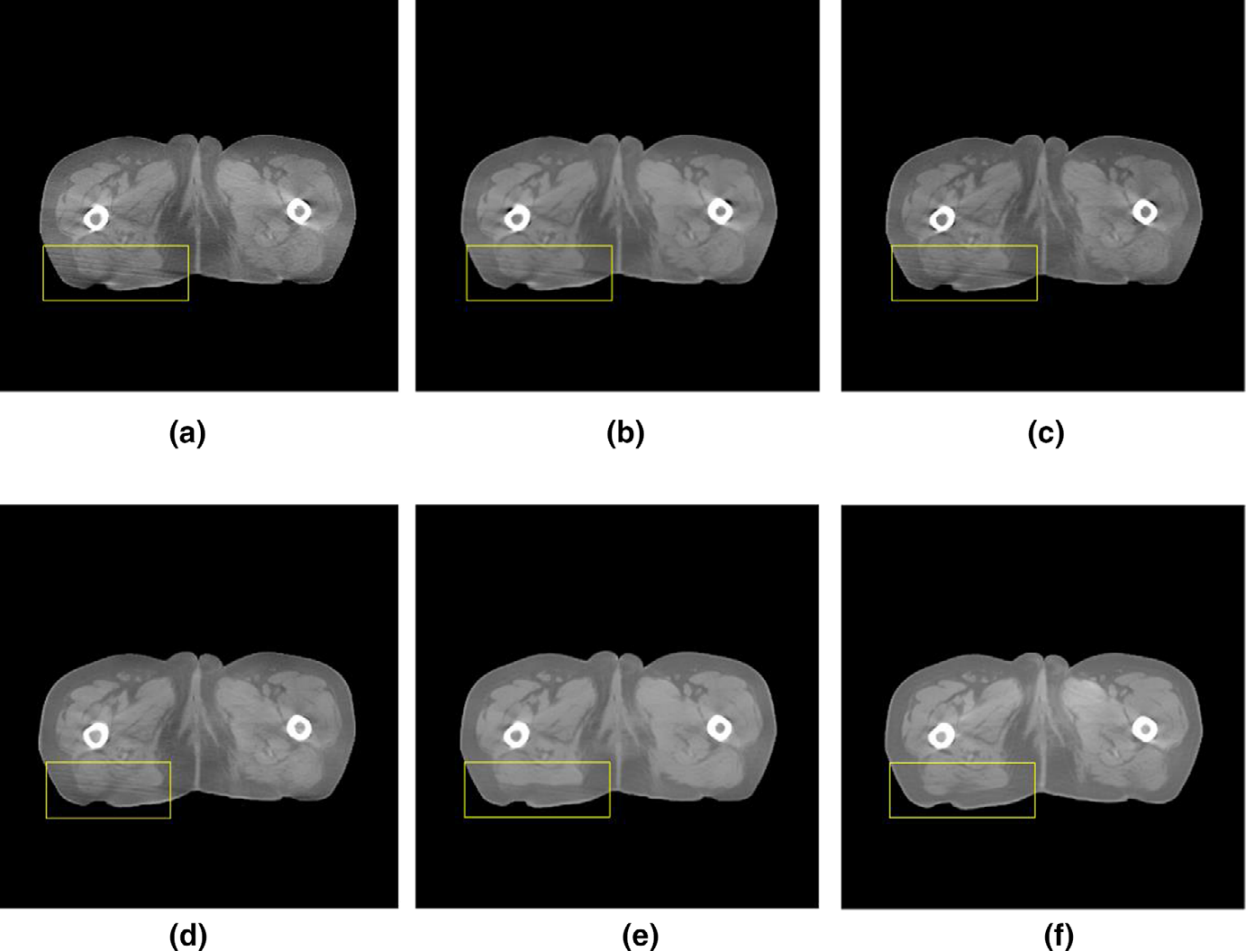
Artifact removal results obtained in optimization process. (a) CBCT, (b) Step 1, (c) Step 2, (d) Step 3, (e) Step 4, and (f) Step 5. Display window [−502, 528].

We take different experiment to find the best parameters of our method, which are simply expressed in Table 4. The experimental results obtained are shown in Fig. 9.

**Table 4:** Parameter optimization process

	Convolutional layers	Dimension	Feature sampling	Loss function
Step 1	17	64	65 × 65	$L\left(G\right)=L_{\mathrm{CX}}^{s}+L_{\mathrm{CX}}^{t}$
Step 2	2	adaptive	65 × 65	$L\left(G\right)=L_{\mathrm{CX}}^{s}+L_{\mathrm{CX}}^{t}$
Step 3	2	adaptive	65 × 65	$L\left(G\right)=L_{\mathrm{CX}}^{s}+L_{\mathrm{CX}}^{t}+{∥G(s)‐t∥}_{2}$
Step 4	2	adaptive	80 × 80	$L\left(G\right)=L_{\mathrm{CX}}^{s}+5L_{\mathrm{CX}}^{t}$
Step 5	5	adaptive	80 × 80	$L\left(G\right)=L_{\mathrm{CX}}^{s}+5L_{\mathrm{CX}}^{t}$

Step 1: The results show that the image quality improved to some extent, but obvious streaking artifacts were still present, as shown in Fig. 9b.

Step 2: The uneven grayscale (cupping artifacts) in the image significantly reduced; however, the stripe artifacts were not removed (Fig. 9c).

Step 3: These changes did not produce the desired results. Fig. 9d shows the results obtained when the L2 constraint was introduced in equation (7).

Step 4: Fig. 9) shows that the streak artifacts were well corrected, and compared with the method used in ^21^, image details were well preserved with no blurring.

Step 5: Comparing Fig. 9e and Fig. 9f, we can be observed that the five‐layer network achieved better artifact correction in the same training time.

The quantitative analysis of the above improvement steps is shown in Table 5 and Table 6.

**Table 5:** MAE (HU) values of different slices during optimization

MAE	CBCT	CT	Step 1	Step 2	Step 3	Step 4	Step 5
Case 1	48.5691	/	49.6123	53.2220	45.9375	41.7456	41.5959
Case 2	43.9188	/	46.6951	48.5677	42.0149	37.5786	37.4898
Case 3	37.0584	/	40.5596	41.5314	35.9510	32.0957	32.1762

**Table 6:** SSIM values of different slices during optimization

SSIM	CBCT	CT	Step 1	Step 2	Step 3	Step 4	Step 5
Case 1	0.8918	/	0.9008	0.8890	0.9012	0.9033	0.9030
Case 2	0.8938	/	0.9032	0.8925	0.9032	0.9062	0.9068
Case 3	0.8893	/	0.8975	0.8897	0.8982	0.9010	0.9028

Table 5 shows that after continuous testing, not only can the artifacts be visually suppressed, but also the MAE index can be more intuitively estimated as the picture quality is significantly improved. Table 6 also shows that the image quality of the CBCT slices processed by the proposed method is closer to the reference CT image.

## 4 discussion and conclusion

CBCT images differ from CT images, and some of the differences remain even after registration. First, the CBCT and CT images of the patients will have different temporal resolutions. Next, slight misalignments and movements can cause differences between the two sets of images. The loss function detailed herein is robust to slight intrinsic differences in the anatomical structure between the CBCT and CT images, which solves the problem of misalignment between the training pairs and yields improved results.

Contextual loss is a loss function based on cosine similarity, which is applied to the feature layer extracted by VGG. The most significant difference from the previously proposed loss functions L1 or L2 loss^33^ is that it does not require the image to be perfectly aligned, thereby allowing local deformation. Perceptual loss^34^ and Gram loss^35^ constrain the similarity of the global high‐frequency features, and these constraints are not very reasonable because the similarity between the images is typically local. The loss function proposed herein can constrain the local features. Furthermore, the loss function is based on semantics and evaluates image similarity based on feature similarity, as opposed to distance. Therefore, the loss function is robust to slight data movements and can address data mismatch problems more effectively.

Furthermore, in this paper, we used the CNN as a feedforward network for scatter artifact correction. This method effectively suppresses the scattering artifacts produced by actual CBCT systems. Moreover, the proposed method can be generalized and applied to different anatomies. In this study, we used the pelvic and thoracic data for testing. Good artifact removal can be achieved, provided that the CBCT images of the anatomies being investigated are collected as training data. We found that method in 21 blurred the detailed texture of the image when we repeated the experiment. The method in 32 is used for deblurring of 2D images. CT is a tomography technique, and the traditional photography technique is a 2D single projection technique. The effect of motion on these two images is different. Therefore, we found that CBCT slices did not obtain satisfactory results using this method, resulting in the loss of some structures. The method in 25 shows good performance in removing artifacts, but the correction on the air cavity needs to be improved. In contrast, it can be seen from Fig. 5b that the proposed method obtained relatively good correction effect on the air cavity. The proposed method preserved the details of the evaluated site, such as textures in the inner contours of the pelvis and chest regions. Moreover, no blurring was introduced into the CBCT slices during artifact removal. The results of the pelvic and thoracic data showed that the proposed method may be useful for removing artifacts in CBCT slices, with a significant improvement in the CT value of the regions of interest. Therefore, the incorporation of our method can effectively reduce the artifacts of CBCT in IGRT and improve the accuracy of dose calculation.

Our proposed method can be further improved using more complex generation networks. Future studies will focus on further investigating and improving our experimental results.

## CONFLICT OF INTEREST

The authors declare no conflict of interest.

## RESEARCH INVOLVING HUMAN PARTICIPANTS AND/OR ANIMALS

This article does not contain any studies with human participants or animals performed by any of the authors.

## INFORMED CONSENT

Informed consent was obtained from all individual participants of the study.
